# Comparative Transcriptome Analyses of Gene Expression Changes Triggered by *Rhizoctonia solani* AG1 IA Infection in Resistant and Susceptible Rice Varieties

**DOI:** 10.3389/fpls.2017.01422

**Published:** 2017-08-17

**Authors:** Jinfeng Zhang, Lei Chen, Chenglin Fu, Lingxia Wang, Huainian Liu, Yuanzhi Cheng, Shuangcheng Li, Qiming Deng, Shiquan Wang, Jun Zhu, Yueyang Liang, Ping Li, Aiping Zheng

**Affiliations:** ^1^Rice Research Institute, Sichuan Agricultural University Chengdu, China; ^2^State Key Laboratory of Hybrid Rice, Sichuan Agricultural University Chengdu, China; ^3^Key Laboratory of Sichuan Crop Major Disease, Sichuan Agricultural University Chengdu, China

**Keywords:** rice, *Rhizoctonia solani* AG1 IA, RNA-seq, differentially expressed gene, defense response

## Abstract

Rice sheath blight, caused by *Rhizoctonia solani*, is one of the most devastating diseases for stable rice production in most rice-growing regions of the world. Currently, studies of the molecular mechanism of rice sheath blight resistance are scarce. Here, we used an RNA-seq approach to analyze the gene expression changes induced by the AG1 IA strain of *R. solani* in rice at 12, 24, 36, 48, and 72 h. By comparing the transcriptomes of TeQing (a moderately resistant cultivar) and Lemont (a susceptible cultivar) leaves, variable transcriptional responses under control and infection conditions were revealed. From these data, 4,802 differentially expressed genes (DEGs) were identified. Gene ontology and pathway enrichment analyses suggested that most DEGs and related metabolic pathways in both rice genotypes were common and spanned most biological activities after AG1 IA inoculation. The main difference between the resistant and susceptible plants was a difference in the timing of the response to AG1 IA infection. Photosynthesis, photorespiration, and jasmonic acid and phenylpropanoid metabolism play important roles in disease resistance, and the relative response of disease resistance-related pathways in TeQing leaves was more rapid than that of Lemont leaves at 12 h. Here, the transcription data include the most comprehensive list of genes and pathway candidates induced by AG1 IA that is available for rice and will serve as a resource for future studies into the molecular mechanisms of the responses of rice to AG1 IA.

## Introduction

Due to their growth characteristics, plants are constantly exposed to different environmental stresses throughout their life cycle, and pathogen infection is a leading constraint on plant growth and productivity (Dodds and Rathjen, 2010). Rice (*Oryza sativa* L.), as the most important food crop for human consumption, provides a stable food supply for more than half of the world’s population. Recently, along with climate change, diseases caused by *Rhizoctonia solani* have severely limited rice production, leading to drastic economic losses each year that threaten food security (Anderson et al., 2004; Marchetti and Bollich, 1991; Lee et al., 2006). *R. solani* is a necrotrophic fungus, and its hyphae grow in close association with host surfaces, forming typical lobate appressoria that can give rise to hyphal aggregates known as infection cushions (Marshall and Rush, 1980). The fungus kills host cells prior to infection by deploying toxins and enzymes that induce cell death (Vidhyasekaran et al., 1997; Brooks, 2007). Despite the huge global losses in rice yield caused by sheath blight, only limited information is available regarding effective control of this disease. In recent years, successful attempts have been made to understand the responses of various rice lines to *R. solani* infection, which have been classified as partial resistance mechanisms but are also referred to as incomplete, quantitative, or horizontal resistance mechanisms (Liu et al., 2013). So far, no rice variety has been found to be highly resistant to *R. solani*, and resistance is conferred solely by the additive effect of non-race-specific resistance QTLs (Pinson et al., 2005; Liu et al., 2009). Control strategies have mainly relied on the application of fungicides at the onset of disease. Our knowledge of the intriguing plant-necrotrophic pathogen interaction established between *R. solani* and rice is still very limited. Therefore, dissecting the molecular mechanism of rice sheath blight resistance would be an important step toward developing novel and effective strategies to control diseases.

As the research area of host and pathogen genomics has rapidly expanded, new technology has provided insights into the processes involved in pathogenesis, host-defense responses, and changes to the structure and composition of the genome associated with diverse host-pathogen interactions (Lai and Mengiste, 2013; Okubara et al., 2014). To date, only a few studies have been conducted to analyze the genome-wide expression profiles of rice after *R. solani* infection and the pathogenic molecular mechanism of pathogens. Venu et al. (2007) detected numerous up- and down-regulated rice genes after infection with *R. solani* using SAGE and microarray analysis. Zhao et al. (2008) found fifty unique cDNA clones and assigned them to five functional categories that had not previously been identified as induced in response to pathogens. Silva et al. (2012) used whole-genome sequencing to identify a total of 333 nsSNPs in resistant lines that were absent in the susceptible group. More than 200 genes with selected nsSNPs were assigned to 42 categories based on gene family/gene ontology. Zheng et al. (2013) identified 25 candidate pathogen effectors based on their functionality and evolution, and three were validated to trigger crop defense responses with the draft genome sequence of *R. solani* AG1 IA that was assembled. Hence, research has revealed that pathogenic determinants exhibit exclusive expression patterns during host infection. Although previous studies have investigated the effects of *R. solani* infection, the molecular mechanisms related to the defense responses of rice against *R. solani* exposure remain poorly understood.

The development and application of second-generation sequencing technology, with its high-throughput ability and low cost, have greatly promoted genomic analysis and gene function research (Wang et al., 2009; Jain, 2012). In recent years, numerous pathogen stress-response genes have been identified in *Arabidopsis thaliana* (Ditt et al., 2006), *O. sativa* (Bagnaresi et al., 2012), *Zea mays* (Skibbe et al., 2010), and *Triticum aestivum* (Yang et al., 2015), and pathogen resistance mechanisms have been explored. To develop a better understanding of the molecular-level variations among rice cultivars after *R. solani* infection, we analyzed the leaf transcriptomes of the rice cultivars TeQing and Lemont after inoculation with AG1 IA. TeQing, a moderately resistant standard rice genotype, and Lemont, a susceptible genotype, were analyzed at 12, 24, 36, 48, and 72 h after AG1 IA inoculation, and Illumina RNA-seq, bioinformatics, qRT-PCR, and transcriptome expression analyses were performed to identify differentially expressed genes (DEGs) and detail how rice responds to AG1 IA. Here, we investigated how these two rice cultivars induce resistance or susceptibility to *R. solani* AG1 IA and whether this response is based on the differential expression of genes involved in different resistance-associated metabolic pathways. To our knowledge, this is the first study to use comparative transcriptome profiling analysis to investigate gene expression patterns in response to AG1 IA infection in resistant vs. susceptible rice genotypes. Unlike SAGE and microarray analysis or whole-genome sequencing analysis, this is the first study to generate extensive transcriptome data in rice after AG1 IA infection. Here, the comparison of RNA-seq data sets from resistant and susceptible genotypes revealed significant differences in expression among genes involved in defense signaling pathways and metabolic pathways and defined the characteristics of transcriptional regulation and identified key genes involved in rice sheath blight resistance.

## Materials and Methods

### Plant Growth Conditions and Pathogen Inoculation

TeQing (a moderately resistant cultivar) and Lemont (a highly susceptible cultivar) seeds were obtained from the Rice Research Institute of Sichuan Agricultural University. The standard strain of *R. solani*, AG1 IA, was kindly provided by Pro. Er-Xun Zhou at South China Agricultural University and has been described previously (Shu et al., 2015). Fourth- and fifth-leaf-stage rice seedlings grown under natural light in a greenhouse (in which the temperature ranged from 20 to 30°C) were inoculated with rice sheath blight fungus, AG1-IA. Before inoculation, the plants were transferred to a growth chamber for adaptive growth. Rice leaves were inoculated with potato dextrose agar (PDA) plugs containing mycelia (Jia et al., 2007). Agar blocks from a 2-day-old mycelia culture grown on PDA (5 mm in diameter to maintain uniform virulence) were placed on the abaxial surface of each leaf piece and covered with plastic wrap to maintain high humidity after inoculation (Supplementary Figure S1A). For the control group, plants were inoculated with agar blocks without fungus. The temperature ranged from 30 to 35°C, the humidity inside the chamber was >80%, and the light was consistent throughout the process. Observation of the symptoms and trypan blue staining patterns was performed to determine the sampling time point (Marshall and Rush, 1980). Here, we chose five time points (12, 24, 36, 48, and 72 h) for RNA-seq.

At the time of harvest, for each treatment group including the control, leaflets were cut from each plant above the leaf sheath. Three biological replicates of lesions were collected randomly from each individual seedling. Leaves inoculated with agar blocks without fungus for 12 h served as the control for each cultivar. 36 samples [T12 (control), T12 h, T24 h, T36 h, T48 h, T72 h, L12 (control), L12 h, L24 h, L36 h, L48 h, and L72 h] were obtained for the present study. Samples for each treatment were harvested separately and immediately frozen in liquid nitrogen and stored at -80°C until RNA extraction for transcriptome sequencing. All samples were sent to Beijing Biomarker Technologies, Inc., for transcriptome sequencing and cDNA library construction.

### Transcript Assembly, Sequence Alignment and Quantification

To obtain expression profiles and predict gene structures, we mapped the pre-processed RNA-seq reads to each reference genome using the Cufflinks program^1^, TopHat2^2^, and Bowtie^3^ (Li et al., 2009; Trapnell et al., 2010; Kim et al., 2013). We used the Nipponbare reference genome MSU7^4^ as a reference sequence. When working with multiple RNA-seq samples, it is necessary to assemble the samples individually and then merge the resulting assemblies together. We used Cufflinks assemblies to reference annotation files and help separate new genes from known ones for use in downstream analyses. Then, the reads from each sample were mapped to the rice genome using TopHat version 2.1.1. The segments of the contiguously unmappable reads are then aligned against these synthetic sequences with Bowtie.

### Screening DEGs

Differential expression was analyzed using the DESeq R package according to the package manual. Raw count data were prepared by a custom Perl script based on the results obtained from eXpress software and were imported into the DESeq framework. Experimental design information was also imported into the DESeq framework to form a count data set. The data were filtered to remove transcripts in the lowest 40% quantile of the overall sum of counts (irrespective of biological condition) to increase the rate of differentially expressed transcript detection. The estimate size factors function was used to estimate the effective library size to normalize the transcript counts. The estimate dispersions function was used to estimate dispersion. The nbinomTest function was used to determine whether there was differential expression between two conditions. False discovery rates (FDRs) were controlled using the Benjamini–Hochberg method at an FDR of 5%. Transcripts per million (TPM) were used to measure the proportion of transcripts in the pool of RNA. Venn diagrams were used to analyze the overlap of differentially expressed transcripts among comparisons.

### Pattern-Mining Based on Time Point and Gene Expression Pattern Analyses

For a control and treatment time series, we designed a series of formulas containing the condition factor, the time factor, and the interaction between the two. Under this premise, we used the likelihood ratio test with a reduced model, which did not contain the interaction terms, to test whether the condition induces a change in gene transcription at any time point after the reference level time point (time 12 h). From this analysis, a total of 4802 DEGs were identified between TeQing and Lemont leaves. We used self-organizing maps (SOMs) to analyze patterns in gene expression (Chang et al., 2010). A matrix of expression values for genes vs. samples was used as input data. Genes with similar expression were self-organized into nearby regions of a supra-hexagonal map. The resulting map was visualized to display the time-specific expression of the genes and was also further partitioned to obtain gene meta-clusters. For each meta-cluster, enrichment analysis was conducted using cluster Profiler packages. Following the analysis, transcripts of DEGs were used as the input for functional enrichment analysis.

### Functional Enrichment Analysis

For functional gene annotation, the obtained unigene sequences were annotated by searching various databases, including the National Center for Biotechnology Information (NCBI), MSU Rice Genome Annotation Project^5^, Gene Ontology (GO)^6^, the Kyoto Encyclopedia of Genes and Genomes (KEGG)^7^, and the China Rice Data Center^8^. Annotation information for homologous genes in these databases was used to represent the annotation of obtained unigenes. In addition, information regarding DEGs of the two rice varieties at different time points was collected from unigene annotations, and these genes were subjected to GO and KEGG significant enrichment analyses to identify the biological functions and related metabolic pathways in which these genes participate.

### Confirmation of the Infection-Responsive Expression Profiles by qRT-PCR

To validate the Illumina sequencing data results, qRT-PCR analysis was performed on 36 RNA samples that were used in the preparation of sequencing libraries. Several genes that were co-expressed in both cultivars were analyzed by qRT-PCR at 12, 24, 36, 48, and 72 h. Specific primers were designed according to individual gene sequences (Supplementary Table S1). cDNA was generated by reverse transcription using the Transcriptor First-Strand cDNA Synthesis Kit (Roche, Indianapolis, IN, United States). Reverse transcription was conducted using a stem-loop RT primer, ST-RT1, as described previously (Li et al., 2016). qRT-PCR experiments were performed on a Bio-Rad CFX96 Real-Time PCR System (Foster City, CA, United States) in accordance with the manufacturer’s instructions. cDNA (2 μl) was amplified with 0.8 μl of specific primers in a total reaction volume of 20 μl, and each reaction was performed four times. We used the UBQ gene as an internal control for data normalization. The 2^ΔΔC_T_^ algorithm was used to calculate the gene expression levels.

## Results

### Early Symptoms of Rice Sheath Blight on Leaves

The major symptoms observed in rice leaves during disease development after AG1 IA infection are shown in Supplementary Figure S1B. Both TeQing and Lemont leaves exhibited noticeable grayish spots approximately 36 h after inoculation. By direct observation, the spots on the Lemont leaves were bigger than those on the TeQing leaves. These symptoms were not apparent at 12 h or 24 h after inoculation on TeQing leaves but appeared on Lemont leaves at 24 h. Trypan blue staining was performed on the leaf tissue near the inoculation site, and an ordinary optical microscope was used to observe hyphae growth. A small number of hyphae appeared at 12 h after inoculation in TeQing leaves, while Lemont leaves had more mycelial biomass than TeQing leaves at 12 h. In TeQing leaves, mycelial biomass was increased at 24 h, and hyphae were proliferative at 48 h. In Lemont leaves, hyphae were denser at 24 h than in TeQing leaves, necrotic lesions and oval-shaped spots with yellow margins were found at 36 h, and the lesion areas were larger and hyphae were more proliferative at 48 h (Supplementary Figure S2). Based on the symptoms observed on the leaves of the two cultivars, we chose 12, 24, 36, 48, and 72 h as time points for RNA-seq.

### RNA-seq Results of Transcriptome Samples

To study the changes in gene expression in TeQing and Lemont leaves infected with AG1 IA at the initial infection stage as described above, we used next-generation sequencing to measure the transcription in rice leaves after AG1 IA inoculation. RNA sequencing data were generated from rice leaf samples at different time points. The transcriptome of each cultivar was analyzed at five time points with three biological replicates for each time point, and Illumina RNA-seq analysis of 36 samples yielded 246.30 Gbp of data and 978,866,157 read pairs (obtained reads) (Supplementary Table S2A). After pre-processing of the reads, we obtained 2,357,278,781 single-end clean reads (total records) (Supplementary Table S2B). The total map ratio ranged from 73.78 to 93.30%, and the unique map ratio ranged from 44.93 to 72.25%. However, many reads could not be mapped to the reference genomes (6.7–26.22%) (Supplementary Table S2B). As these datasets were generated from different rice cultivars and at different time points, expression levels in all samples were calculated uniformly, as described previously (Wang et al., 2014). We used sample-to-sample correlation analysis for the data exploration analysis. The overall relatedness of the transcriptome at different times was determined by a cluster dendrogram generated for the TeQing and Lemont samples (Supplementary Figure S3). For all samples, the three biological replicates (TeQing and Lemont) showed good correlation, and the transcriptome data were closely related at each time point. Also PCA was used to assess the similarity between samples across conditions using separate samples from the different biological conditions according to the first principal component (PC1) and the second principal component (PC2), meaning that the biological variability between two groups was the main source of variance in the data (Supplementary Figure S4). This may occur for several reasons, such as RNA contamination between samples (even between biological replicates) or slight differences between library concentrations, since they may be difficult to distinguish with high precision.

### Differential Gene Analysis of TeQing and Lemont Leaves at Different Time Points

To determine which genes exhibit expression changes and at what stage these changes occur, comparisons between the rice cultivars, with controls, were performed. A comparison between the two varieties yielded data regarding differentially expressed rice genes at different time points after AG1 IA inoculation (**Table 1**). After AG1 IA inoculation, the number of up-regulated genes exceeded the number of down-regulated genes, and genes were more up-regulated in Lemont leaves than in TeQing leaves at all time points. At 12 h, 4,205 DEGs (2,348 up-regulated, 1,857 down-regulated) were identified in Lemont leaves, compared with 1,676 DEGs in TeQing leaves (1,021 up-regulated, 655 down-regulated), suggesting that Lemont leaves are more susceptible to AG1 IA than TeQing leaves and that the infection pressure is greater on Lemont plants than on TeQing plants. The number of DEGs in Lemont leaves was highest at 24 h (7,041 DEGs; 4,781 up-regulated, 2260 down-regulated) and 48 h (5,920 DEGs; 4,201 up-regulated, 1,719 down-regulated) after AG1 IA inoculation and exceeded the number of DEGs in TeQing leaves at the same time points. The data suggest that after AG1 IA infection, differential gene expression was induced to a greater extent in the susceptible cultivar (24 and 48 h) than in the resistant cultivar. As for the differences observed at 24 and 48 h compared with other time points, we speculate that primary pathogen colonization occurred within 24 h based on the appearance of symptoms and on hyphae growth observed under a microscope. The pathogen then established re-infection, causing the degree of infection to be enhanced at 48 h.

**Table 1:** Statistics of differentially expressed genes.

Combination	DEG set name	Up-regulated	Down-regulated	All DEGs
TCK_vs_T12	T12	1021	655	1676
TCK_vs_T24	T24	3957	1350	5307
TCK_vs_T36	T36	789	692	1481
TCK_vs_T48	T48	2473	650	3123
TCK_vs_T72	T72	1095	211	1306
LCK_vs_L12	L12	2348	1857	4205
LCK_vs_L24	L24	4781	2260	7041
LCK_vs_L36	L36	2025	1034	3059
LCK_vs_L48	L48	4201	1719	5920
LCK_vs_L72	L72	1543	483	2026

*TCK_vs_T12, TCK_vs_T24, TCK_vs_T36, TCK_vs_T48, and TCK_vs_T72 represent the comparison of TeQing leaves infected with AG1 IA for 12, 24, 36, 48, or 72 h and TeQing control leaves after 12 h, respectively; the same follows for LCK_vs_L12, LCK_vs_L24, LCK_vs_L36, LCK_vs_L48, and LCK_vs_L72. Unigenes with a false discovery rate (FDR) no greater than 0.01 and reads per kb per million reads (RPKM) between samples of no less than 2 [fold change (FD) ≥ 2] were considered differentially expressed genes (DEGs).*

**FIGURE 1:**
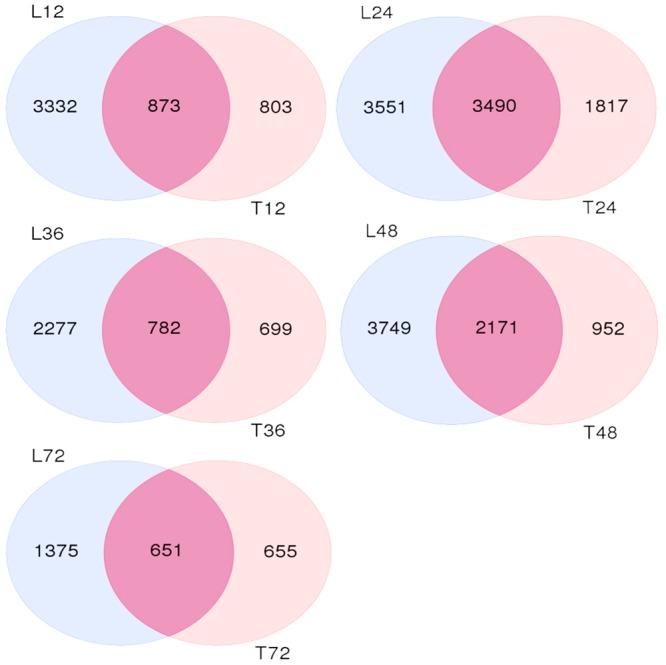
Venn diagram of differentially expressed genes found in both rice cultivars after AG1 IA infection at the same time points. L12, L24, L36, L48, and L72 denote differentially expressed gene sets obtained from Lemont leaves at 12, 24, 36, 48, and 72 h after AG1 IA infection, respectively, compared to control leaves at 12 h; the same is shown for T12, T24, T36, T48, and T72.

**FIGURE 2:**
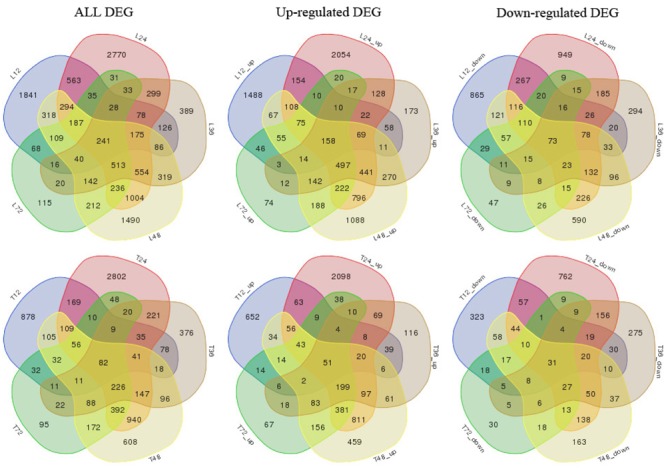
Venn diagram of differentially expressed genes in both rice cultivars at different time points after AG1 IA inoculation. T12, T24, T36, T48, and T72 denote differentially expressed gene sets obtained by comparing TeQing samples at 12, 24, 36, 48, and 72 h after AG1 IA inoculation, respectively, with the control sample at 12 h; L12, L24, L36, L48, and L72 denote differentially expressed gene sets obtained by comparing Lemont samples at 12, 24, 36, 48, and 72 h after AG1 IA inoculation, respectively, with the control sample at 12 h. All DEGs, all differentially expressed genes; Up-regulated DEGs, up-regulated genes; Down-regulated DEGs, down-regulated genes.

We also analyzed both cultivar controls and samples at 12, 24, 36, 48, and 72 h after AG1 IA inoculation. The samples were subjected to multi-group differential analysis to identify DEGs in rice associated with response to AG1 IA infection. DEG sets at the same time points in both cultivars (L12–T12, L24–T24, L36–T36, L48–T48, and L72–T72) were compared (**Figure 1**). At 24 and 48 h after AG1 IA inoculation, 3,490 and 2,171 DEGs were identified in both cultivars, which was more than at other time points. In addition, the time-dependent expression of DEGs was analyzed by comparing the expression at different time points in each cultivar (T12–T24–T36–T48–T72 h or L12–L24–L36–L48–L72 h) (**Figure 2**). In TeQing leaves, 82 common DEGs exhibited sustained expression at 12, 24, 36, 48, and 72 h after inoculation (51 up-regulated; 31 down-regulated). At 12 h, 878 specific DEGs were identified (652 up-regulated; 323 down-regulated). At 24 h, 2,802 specific DEGs were identified (2,098 up-regulated; 762 down-regulated), and 608 specific DEGs were identified (459 up-regulated; 163 down-regulated) at 48 h. In Lemont leaves, 241 genes had sustained expression at 12, 24, 36, 48, and 72 h after inoculation (158 up-regulated; 73 down-regulated). At 12 h, 1,841 specific DEGs were identified (1,488 up-regulated; 865 down-regulated). At 24 h, 2,770 specific DEGs were identified (2,054 up-regulated; 949 down-regulated). At 48 h, 1,490 specific DEGs were identified (1,088 up-regulated; 590 down-regulated). Overall, from 12 to 72 h after AG1 IA infection, more continuously expressed genes were observed in Lemont leaves than in TeQing leaves.

To identify the functional distribution of unigenes, we used GO annotations to classify the enriched DEGs between the control and AG1 IA-treated groups. These genes were involved in all aspects of biological activity, and intriguingly, the distribution of annotated unigenes in the two cultivars showed highly similar patterns (Supplementary Figure S5). All the unigenes annotated in the GO database were classified into three main categories. Within biological processes, “photosynthesis,” “generation of precursor metabolites and energy,” “growth,” and “cell death” were the four most common GO terms. For the cellular components category, most of the unigenes were related to “peroxisome,” “nucleolus,” and “Golgi apparatus.” In the molecular functions category, unigenes were mostly involved in “binding,” followed by “nuclease activity” and “enzyme regulator activity.” These highly enriched GO terms mainly refer to the maintenance of the basic regulation and metabolic functions of rice after AG1 IA infection. Additionally, GO terms related to special disease resistance functions, such as “cell death” in the biological process category and “oxygen binding” in the molecular function category, were found. From the results, we selected GO terms related to pathogen resistance to analyze the differences between TeQing and Lemont leaves. For example, genes related to photosynthesis were maintained at a relatively stable level in TeQing leaves compared with Lemont leaves in the early stage of infection. Genes related to the generation of precursor metabolites and energy exhibited a larger increase in expression in TeQing leaves than in Lemont leaves at 24 h. For genes annotated by the GO terms “cell death,” “peroxisome,” and “oxygen binding,” we also found that the change in expression was larger in TeQing leaves than in Lemont leaves. From the GO terms, we obtained an overview of ontology content and revealed the global biological activities occurring in the TeQing and Lemont leaves after AG1 IA infection. Our results suggest that at early time points in the rice-AG1 IA interaction, plant defense-related genes are more active in Lemont leaves than in TeQing leaves. Thus the resistant and susceptible cultivars had specific reactions in response to AG1 IA infection.

### Analysis of the Dynamic Gene Expression Patterns of Both Rice Cultivars

**FIGURE 3:**
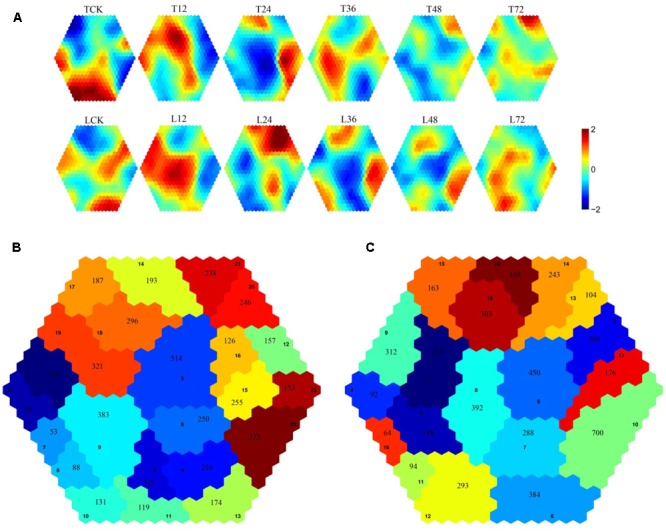
**(A)** Temporal expression patterns of 4802 genes in TeQing and Lemont leaves after AG1 IA inoculation. **(B)** Twenty-three robust clusters (1–23) were identified for TeQing by SOM clustering; the separate clusters are color-coded. **(C)** Nineteen robust clusters (1–19) were identified for Lemont by SOM clustering; the separate clusters are color-coded. The gene numbers are shown in the separate clusters.

**FIGURE 4:**
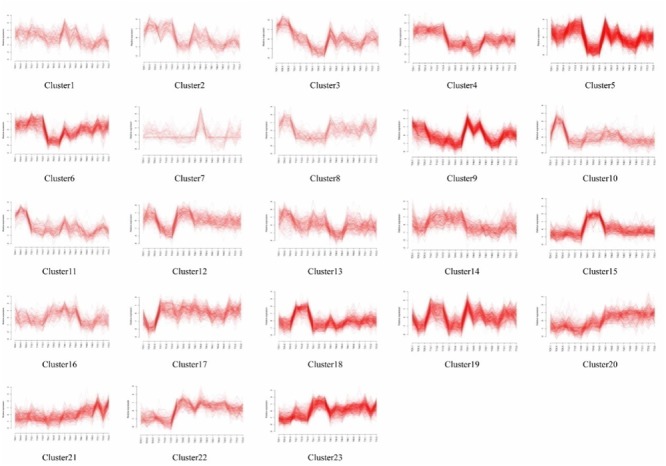
Temporal expression profiles for the 23 clusters of 4802 genes in TeQing leaves.

**FIGURE 5:**
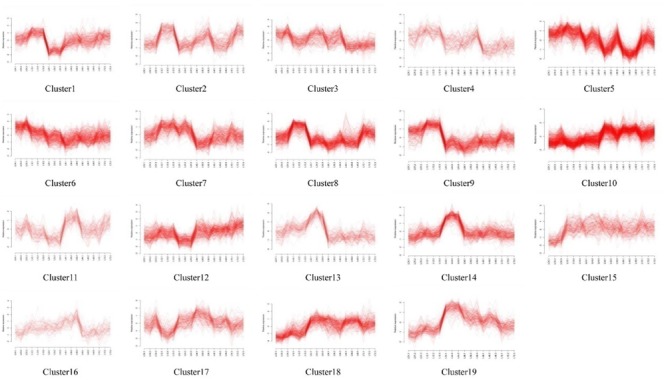
Temporal expression profiles for the 19 clusters of 4802 genes in Lemont leaves.

Self-organizing maps were used to identify the dynamic changes among the 4,802 common DEGs at different time points to compare the two rice varieties (Supplementary Table S3), and multi-group expression pattern clustering analysis was performed. Each component plane shows the hexagonal variation in gene expression [log2 (pond/upland)] at one time point, using a color gradient from blue to red to indicate up- and down-regulation (see the colored bar). Gene sets generated using the SOM algorithm were projected to different small grids, and adjacent gene grids have similar expression patterns (**Figure 3A**). The lattices are clustered according to gene expression levels, and lattices belonging to the same cluster have the same color (**Figures 3B,C**). Finally, the characteristic values of the gene expression levels in each lattice were extracted and are presented in **Figures 4, 5** to show the expression levels of the lattice genes. GO classification analysis was performed to determine whether the differentially regulated genes in each cluster were significantly associated with a specific biological process, molecular function or cellular component (Supplementary Tables S4, S5). From the SOM, we found that both the TeQing and Lemont DEGs were primarily concentrated during the early stage of infection (12 and 24 h). The two rice cultivars exhibited very different defense patterns after AG1 IA infection. We selected several clusters to further analyze the differential expression patterns in the two rice cultivars.

In the patterns of cluster No. 17 in TeQing and cluster No. 15 in Lemont, the levels of DEGs exhibited sustained induction after AG1 IA inoculation. The up-regulation of these genes began within 12 h and persisted throughout the 72 h experimental period. The sustained accumulation of this gene type suggests important biological significance in response to AG1 IA. We collected GO annotations to classify the DEGs identified in TeQing and Lemont leaves. Cluster 17 in TeQing was enriched in genes involved in cell death, responses to extracellular stimuli, peroxisomes, the Golgi apparatus, and enzyme regulation and signal transduction activities. Cluster No. 15 in Lemont mainly included genes involved in the generation of precursor metabolites and energy, the regulation of cell death, the Golgi apparatus, lipid binding and motor activity.

In the patterns of cluster No. 11 in TeQing and cluster No. 6 in Lemont, we found that the level of induction of the DEGs decreased steadily relative to the original level and reached a minimum after 72 h of plant–pathogen interaction. The gene expression levels in cluster No. 11 in TeQing showed a steeper decline than those in cluster No. 6 in Lemont. GO analysis showed that cluster No. 11 in TeQing was enriched in genes related to the nucleolus and that cluster No. 6 in Lemont was mainly enriched in genes involved in the generation of precursor metabolites and energy, cell death, photosynthesis, cellular homeostasis, nucleolus, oxygen binding and nuclease activity.

In the patterns of cluster No. 12 and cluster No. 19 in TeQing, the levels of DEGs decreased at 12 h and then peaked at 24 h in cluster No. 12. Cluster No. 19 showed an opposite expression pattern: the levels were high at 12 h but decreased at 24 h. Based on the GO classifications, we found that in the two dynamic expression patterns, cluster No. 12 was enriched in genes involved in the generation of precursor metabolites, cellular homeostasis, nucleolus, and enzyme regulator activity, and cluster No. 19 was mainly enriched in genes related to the regulation of gene expression, epigenetics, the cell cycle, nucleoplasm, Golgi apparatus, cytoskeleton, nucleolus, signal transduction activity and oxygen binding.

Interestingly, the genes of Cluster No. 20 and No. 21 in the moderately resistant cultivar TeQing were continuously up-regulated after AG1 IA inoculation. The expression levels of these genes peaked at 72 h after infection treatment, and these genes were involved in cell death, responses to extracellular stimuli, cell communication, cellular homeostasis, the cytoskeleton and oxygen binding. In Cluster No. 10 in Lemont, the DEGs up-regulated at 36 h were analyzed using GO enrichment. This cluster was significantly enriched in genes associated with the generation of precursor metabolites and energy, cellular homeostasis, cell death, the Golgi apparatus and oxygen binding.

### Analysis of Metabolic Pathways after AG1 IA Inoculation

To further investigate the biological functions and interactions of differentially regulated transcripts specific to TeQing and Lemont leaves after AG1 IA inoculation, pathway-based analysis was conducted using KEGG. To further analyze the differences between the two rice cultivars, we examined the KEGG categories with *p*.adjust < 0.01 that were used to classify up-regulated genes into different biological processes. These categories are presented in Supplementary Tables S6, S7, and the unigenes involved in resistance-associated metabolic pathways were included.

At 12 h, the highly represented pathways in TeQing leaves compared with Lemont leaves were glycine, serine and threonine metabolism (*p*.adjust = 2.72E-05), phenylalanine, tyrosine and tryptophan biosynthesis (*p*.adjust = 2.72E-05) and phenylpropanoid biosynthesis (*p*.adjust = 0.001256). At 24 h, the primary pathways enriched in TeQing leaves were phenylalanine, tyrosine and tryptophan biosynthesis (*p*.adjust = 8.54E-10), alpha-linolenic acid metabolism (*p*.adjust = 2.25E-08), phagosome (*p*.adjust = 6.37E-08), and plant–pathogen interaction (*p*.adjust = 1.66E-06). In Lemont leaves, the phenylalanine, tyrosine and tryptophan biosynthesis (*p*.adjust = 3.67E-10) and plant–pathogen interaction (*p*.adjust = 1.77E-09) pathways were significantly enriched. In addition, we found that the valine, leucine and isoleucine degradation (*p*.adjust = 0.003265), phenylalanine metabolism (*p*.adjust = 0.00423) and phenylpropanoid biosynthesis (*p*.adjust = 0.000247) pathways were also enriched in TeQing leaves at 24 h.

**FIGURE 6:**
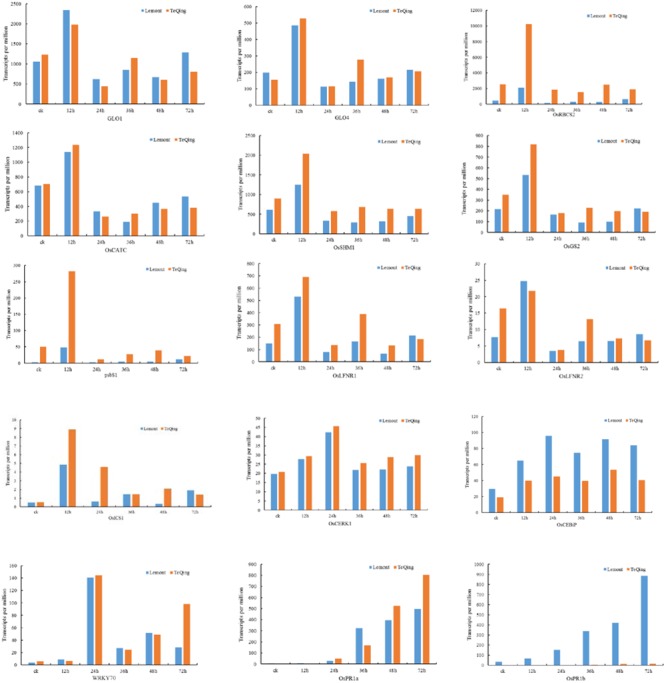
Expression of genes involved in photosynthesis, photorespiration and other processes in TeQing and Lemont leaves based on transcripts per million (TPM).

To further analyze the effects of related metabolic pathways on plant resistance, TPM was used to compare the expression of key genes involved in metabolic pathways between TeQing and Lemont leaves (**Figure 6**). In addition to photosynthesis metabolism, PsbS was rapidly increased in TeQing leaves after AG1 IA treatment. Additionally, we found that OsLFNR1 and OsLFNR2 were both increased at 12 h in the two rice varieties. OsLFNR1 was more highly expressed in TeQing leaves than in Lemont leaves. Based on the finding that glycine, serine and threonine metabolism (*p*.adjust = 2.72E-05) and glyoxylate and dicarboxylate metabolism (*p*.adjust = 2.72E-05) were induced in TeQing leaves at 12 h, we examined the expression of genes that were closely related to photorespiration. GLO1 and GLO4 were both increased in TeQing and Lemont leaves, and GLO4 was more highly expressed in TeQing leaves at 12 h. In addition, other genes, such as serine hydroxymethyl transferase (OsSHM1), catalase (OsCATC), glutamine synthetase (OsGS2) and ribulose-1,5-bisphosphate carboxylase/oxygenase small subunit (OsRBCS2), that participate photorespiration were found to be increased at 12 h. Based on the observation that plant-pathogen interaction pathways were induced at 24 h, we found that rice LysM receptor-like kinase (OsCERK1), rice chitin receptor chitin oligosaccharide elicitor-binding protein (OsCEBiP) and OsFLS2 were expressed in TeQing and Lemont leaves. These results were corroborated by the fact that the expression of CEBiP and CERK1 was maximal at 24 h. Most of the genes related to calcium signaling were down-regulated at 12 h but increased at 24 h. Additionally, mitogen-activated protein kinase (MAPK), WRKY, and pathogenesis-related genes as well as other genes were found to be involved in this metabolic pathway.

**FIGURE 7:**
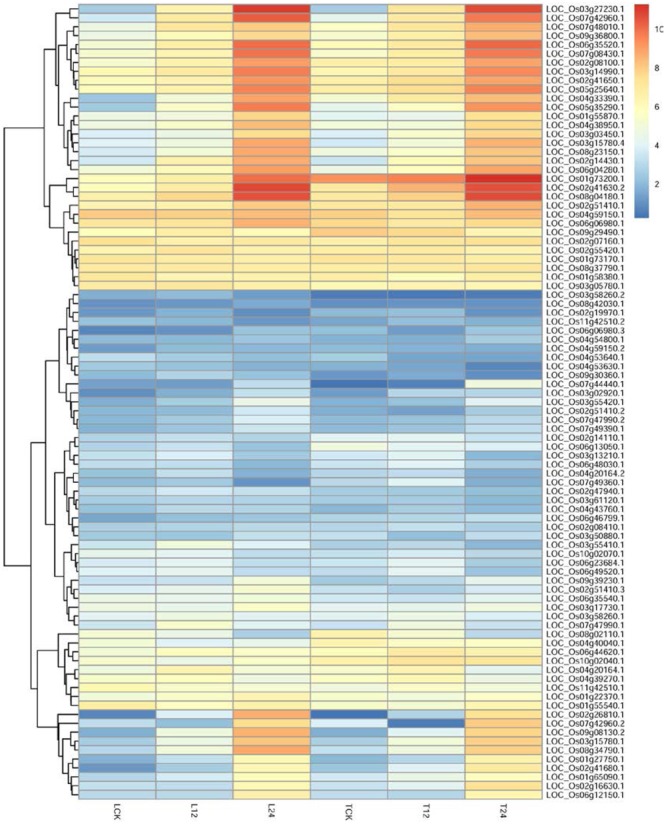
Detailed expression profiles of genes related to phenylalanine metabolism pathways at 12 and 24 h.

**FIGURE 8:**
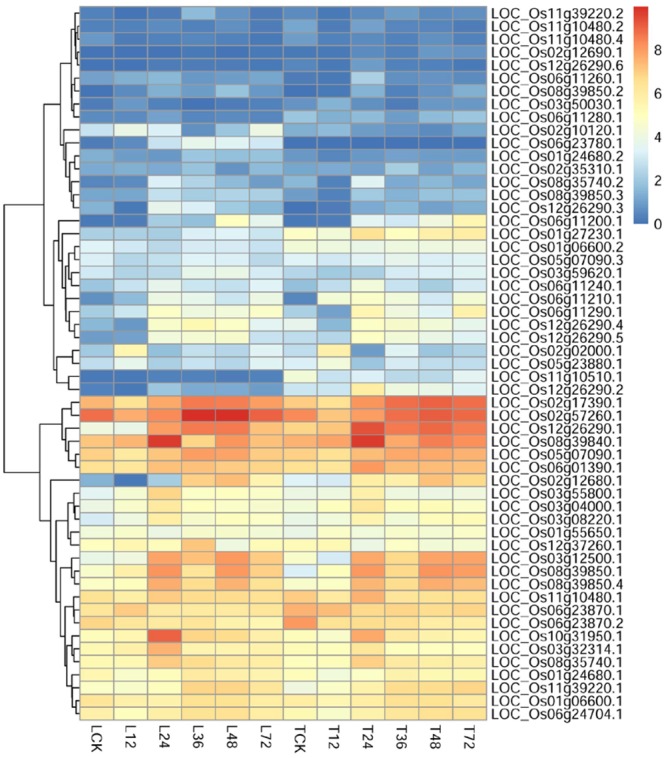
Detailed expression profiles of genes related to alpha-linolenic acid metabolism.

To identify potential regulatory genes tightly related to phenylalanine metabolism at 12 h, we identified DEGs by comparing the expression changes between the two varieties. Of the genes involved in phenylalanine metabolism that were expressed at 12 h, more were expressed at a higher level in TeQing leaves than in Lemont leaves; the difference between the two varieties can be seen in the heatmap (**Figure 7**). Genes associated with the KEGG term alpha-linolenic acid metabolism, which is an important precursor of jasmonic acid (JA), were found in TeQing and Lemont leaves. Corresponding genes were identified, and their expression is shown in **Figure 8**. These genes included 12-oxophytodienoic acid reductase, 3-ketoacyl-CoA thiolase, lipoxygenase, allene oxide cyclase, phospholipase, and cytochrome P450, which all participate in JA biosynthesis (Wang et al., 2000; Zhang et al., 2005; Schaller and Stintzi, 2009). Because more than 90% of salicylic acid (SA) is synthesized via the isochorismate synthase (ICS) pathway (Wildermuth et al., 2001), we used OsICS1 to analyze the changes in SA synthesis in the two cultivars. OsICS1 was expressed in both TeQing and Lemont leaves, exhibiting maximum expression at 12 h and then decreasing. However, TeQing leaves expressed more OsICS1 than Lemont leaves at 12 h (**Figure 6**).

Our results revealed that the metabolic pathways of both varieties participate in similar mechanisms after AG1 IA infection, but in the initial period of pathogen infection, TeQing leaves exhibited an earlier response to pathogen infection than Lemont leaves. However, during pathogen infection, genes involved in alpha-linolenic acid metabolism were up-regulated in Lemont leaves after 24 h compared with TeQing leaves, and related resistance-associated metabolic pathways were significantly enriched in Lemont leaves. Comparing the expression of genes involved in phenylalanine metabolism and alpha-linolenic acid metabolism in TeQing and Lemont leaves indicated a time-shift in the expression patterns. Further research into DEGs involved in metabolic pathways is needed to understand the mechanisms underlying AG1 IA resistance.

### Quantitative RT-PCR (qRT-PCR) Validation of DEGs

The RNA-seq data were validated by quantitative reverse transcription PCR (qRT-PCR) analysis. The rice pathogenesis-related proteins PR1, PR5, and PR10 were selected for validation (Maruthasalam et al., 2007; Wu et al., 2011), as was WRKY72, a member of the *WRKY* gene superfamily in rice whose expression is reportedly induced expression during the infection of *Magnaporthe oryzae, Ustilaginoidea virens* and *X. oryzae* pv. *oryzicola* (Chao et al., 2014). In addition, several genes involved in metabolic pathways were also selected for validation. The *C*t values obtained were normalized to those of the internal control, and the fold change upon AG1 IA infection was calculated for both rice cultivars. In addition, the fold change of transcripts in infected TeQing and Lemont leaves with respect to control conditions were also calculated. The differential expression values of all selected transcripts obtained using qRT-PCR analysis were plotted along with the RNA-seq data and their patterns were compared (Supplementary Figure S6). The qRT-PCR analyses showed trends in expression that were consistent with those found by RNA-seq, suggesting that the Illumina data are relatively reliable.

## Discussion

In recent years, RNA-seq has been widely used in the screening of different genes, functional gene mining and the analysis of molecular mechanisms of disease resistance in plant-pathogen interactions (Kawahara et al., 2012). It was previously reported that differential expression analysis at the isoform level can provide a more comprehensive overview of gene regulation than gene-level expression patterns (Nakashima and Yamaguchi-Shinozaki, 2009). Here, using two different types of rice cultivars, we revealed that the differential regulation of gene expression exhibited a very different defense pattern between cultivars and that TeQing leaves respond more quickly during the initial infection stage. These data show that in both TeQing and Lemont leaves, DEGs were primarily concentrated in the early stages of infection. Our results may facilitate the discovery and annotation of important genes in the defense response, physiology and metabolism of plants and provide comprehensive infection-responsive expression profiles for the rice response to AG1 IA. Further investigation of these differentially regulated genes will provide insights into the differential pathogen responses of different rice cultivars.

### Gene Expression Changes in Response to AG1 IA Infection

Previous studies have shown that in susceptible plants, a slow response and weaker defense signals allow pathogens to travel through the tissue and damage the plant. Such molecular changes affect later alterations in plant appearance, physiology and biochemistry (Que et al., 2014). Research has shown that the resistance of a plant to necrotrophic fungi is based on its ability to counteract the toxic effects of reactive oxygen species (ROS), which may involve the production of antimicrobial metabolites, such as phytoalexins, and the induction phytohormone-regulated signaling pathways, predominantly the JA and cell death pathways (Glazebrook, 2005). Various numbers of GO terms were enriched in each rice cultivar after AG1 IA infection. The major differences between TeQing and Lemont plants were observed in the timing of host recognition and in the speed and effectiveness of the defense response to AG1 IA invasion. In addition, the number of genes expressed in Lemont leaves was greater than that in TeQing leaves throughout AG1 IA infection, which may indicate that the different rice cultivars activate different mechanisms upon AG1 IA infection. It has been suggested that during plant–pathogen interactions, the role of primary metabolism is to support the cellular energy requirements for plant defense responses to establish a favorable energy balance for defense (Kangasjärvi et al., 2012; Andolfo and Ercolano, 2015). Through various metabolic processes, plants produce many metabolites that could improve disease resistance, and these metabolites form an important material basis for plant resistance systems (Rojas et al., 2014). Here, based on our KEGG analysis, we compiled evidence from the literature to show that upon exposure to pathogens, plants induce genes associated with primary metabolic pathways. Transcriptome sequencing analysis showed that most of the genes and pathways induced in both cultivars were similar. This is consistent with previous data suggesting that after pathogens infect plants, many metabolic pathways are affected, and gene expression in the transcription network is disturbed (Bolton et al., 2008; Coram et al., 2008).

### Photosynthesis and Photorespiration

The defense response requires energy, and this causes an increased demand for photosynthesis, as photosynthesis provides a carbon source for the synthesis of defense compounds (Hammerschmidt, 1999). Previous studies have revealed the link between the defense response and photosynthesis (Kerchev et al., 2012). Non-photochemical quenching (NPQ) is an essential process that protects plants from excess light energy and resulting damage from ROS formation (Müller et al., 2001). Göhre et al. (2012) found that activation of defense mechanisms by pathogen-associated molecular patterns (PAMPs) could lead to a rapid decrease in NPQ and that NPQ also influences several PAMP-triggered immune response processes. In addition, they proposed that NPQ might play a role in positively regulating PAMP-triggered immunity (PTI). PsbS is a central player involved in NPQ and controls the macro-organization of photosystem II complexes (Niyogi et al., 2005; Kereiche et al., 2009). The expression of PsbS observed here suggests that NPQ might be increased upon AG1 IA infection. The ferredoxin-thioredoxin system is a well-known mechanism that activates light-driven metabolic reactions in chloroplasts. Light-driven redox chemistry also provides plants with a mechanism for generation of ROS, which are key players in the relay of stress signals in photosynthetic tissues (Buchanan and Balmer, 2005). A previous study showed that transient increases in the levels of ROS in chloroplasts have vital signaling roles in the initiation of immune reactions upon attempted infection in different cell types (Gechev et al., 2006). In addition to ROS formation, nitric oxide (NO) generation and the induction of SA and JA biosynthesis occur in chloroplasts and contribute to the specificity of immune reactions (Boller and He, 2009). Here, we found that genes related to photosynthesis were increased at 12 h and then decreased. As previous study suggested that the decrease in photosynthesis after infection might be an active mechanism of the plant defense program that limits the carbon source availability for the pathogen or that it may result from the prioritizing of metabolic processes that contribute to plant defense responses (Bolton, 2009).

Photorespiration, an important process that produced H_2_O_2_, is critical in the plant defense response and regulates the cell redox state. Research has shown that photorespiration reduces the Mehler reaction rate, mitigates the potential hazards of ROS and provides more effective protection to the photosynthetic apparatus than the Mehler reaction (Wu et al., 1991). H_2_O_2_ can trigger allergic reactions in plants, protecting healthy cells from infection through the programmed cell death of infected cells. The increased expression of genes involved in photorespiration metabolism can result in an increased H_2_O_2_ content in plants, which may improve plant disease resistance. One example is glycolate oxidase (GO), a key enzyme involved in plant photorespiration in the peroxisome. Research has found that GO is an alternative source for the production of H_2_O_2_ during both gene-for-gene and non-host resistance responses (Rojas and Mysore, 2012). In addition, serine hydroxymethyltransferase (SHMT) is an essential component of photorespiration. A previous study found that an osshm1 mutant exhibited chlorotic lesions and a lethal phenotype (Wang et al., 2015). Lin et al. (2012) found that OsCATC could increase hydrogen peroxide in leaves, which consequently promoted NO production and led to cell death. Thus, based on our gene expression results, we propose that photorespiration may play an important role after AG1 IA infection. Here, we have described the potential effects of photosynthesis and photorespiration on rice sheath blight resistance; however, further study is necessary to investigate how photosynthesis and photorespiration regulate sheath blight resistance.

### Plant–Pathogen Interactions

Plants have established a series of defense mechanisms against pathogens during their co-evolution. The innate plant immune system can be generally thought of as having two layers. The first layer of immunity, known as PTI, is based on the recognition of PAMPs by cell surface pattern-recognition receptors, which confers resistance to most pathogenic microbes. The second layer of immunity primarily relies on the recognition of pathogen-secreted effectors by plant-specific resistance proteins in direct or indirect ways, which leads to race-specific effector-triggered immunity (ETI) (Jones and Dangl, 2006). Chitin, a major component of fungal cell walls, is a representative PAMP. When a plant is attacked by a fungal pathogen, the pattern-recognition receptors located on the plasma membrane can recognize chitin and chitin oligosaccharide and trigger innate immune responses (Lenardon et al., 2010). CEBiP and CERK1 have been identified as critical components in chitin signaling in rice and Arabidopsis, respectively (Kaku et al., 2006; Lee et al., 2014). Here, we found that OsCERK1 exhibited a completely different trend in TeQing leaves at 12 h, and we believe that OsCERK1 may participate in disease resistance in several different ways. Other genes, such as OsPti1a, which has been shown to have an important role in regulating defense signaling for both R gene-mediated and basal resistance (Takahashi et al., 2007; Matsui et al., 2015) (Supplementary Table S8), increased in both TeQing and Lemont leaves but exhibited a more pronounced expression change in TeQing leaves at 24 h.

Calcium, as an essential messenger, participates in the regulation of physiological metabolism through plant signal transduction pathways. Research has found that Ca^2+^ participates in oxidative burst signal transduction, regulates the expression of PR genes and mediates hypersensitive cell death (Torres and Dangl, 2006; Ogasawara et al., 2008; Du et al., 2009). Here, we observed the expression of genes involved in calcium-dependent signaling in plant–pathogen interactions in response to AG1 IA (Supplementary Table S8). One example is OsCPK10, a crucial regulator in plant immune responses that may regulate disease resistance by activating both SA- and JA-dependent defense responses (Fu et al., 2013). We found that most of the genes in the calcium signaling pathway were down-regulated at 12 h and up-regulated at 24 h. In addition, the degree of gene expression variability was higher in TeQing leaves than in Lemont leaves. Further research is needed to determine the effects of calcium on sheath blight resistance. Kinase cascades of the mitogen-activated protein kinase (MAPK) class play a critical role in the defense signaling evoked by the recognition of microbial-associated molecular patterns (MAMPs) that regulate several defense responses (Pitzschke et al., 2009). As shown in Supplementary Table S8, genes such as OsMKK1, OsMKK4, and OsMKK6 as well as other stress receptors exhibited changes in transcript levels in response to AG1 IA based on plant-pathogen interactions. Research has shown that OsMKK4-OsMPK3/OsMPK6 contribute substantially to the chitin elicitor-inducible biosynthesis of diterpenoid phytoalexins by regulating the expression of their biosynthetic genes (Yamane, 2013).

Members of the WRKY family are widely involved in plant defense responses, including positive and negative regulation of disease resistance (Pandey and Somssich, 2009). Here, we found that three WRKY transcription factors (WRKY24, WRKY53, and WRKY70) were expressed in both rice cultivars and that they were expressed at higher levels in TeQing leaves than in Lemont leaves. The expression level of WRKY70 increased at 12 h and reached a maximum at 24 h (**Figure 6**). Research has found that WRKY70 acts as an activator of SA-induced genes and a repressor of JA-responsive genes. The level of WRKY70 transcripts therefore reflects the cellular SA/JA balance and determines which type of response is favored (Li et al., 2004). The PR1 gene is often used as a molecular marker of disease resistance. Research has shown that the expression of OsPR1a and OsPR1b increases after infection with *Xanthomonas oryzae* pv. *oryzae* (Xoo) and *Magnaporthe oryzae* (Wu et al., 2011). We found that the expression of these two genes in the two varieties was completely opposite (**Figure 6**). We believe that OsPR1a and OsPR1b may function in different pathways after AG1 IA infection.

### Changes in Phenylalanine Compounds

Previous studies have shown that in the secondary metabolism of plant disease resistance, phenylpropanoid metabolism is an important metabolic pathway; this pathway includes flavonoids, stilbenes, monolignols, and various phenolic acids and participates in the formation of secondary resistance metabolites (Jones, 1984), such as phytoalexin (Dixon and Fuller, 1976), lignin (Legrand et al., 1976) and phenolic compounds (Glazener, 1982). KEGG results showed that phenylalanine metabolism plays an important role in TeQing leaves throughout the infection period, especially at 12 h. Through gene analysis, a series of peroxidase (POD) genes and phenylalanine lyase (PAL) genes were found to be stimulated after AG1 IA inoculation. A previous study found that POD participates in the polymerization of monolignols into lignin and the reinforcement of the cell wall after pathogen attack (Marjamaa et al., 2009). Research has also shown that POD plays an important role in generating H_2_O_2_ as part of the defense response and confers resistance to a wide range of plant pathogens (Bindschedler et al., 2006). PAL plays a critical role in the phenylpropanoid pathway and has been reported to be responsive to pathogen attack (Huang et al., 2010). Research has shown that after pathogen infection, the activity of PAL exhibits dynamic changes over time (Nagarathna et al., 1993) and that PAL activity increased upon the development of HR in plant disease resistance (Southerton and Deverall, 1990). Here, nine differentially expressed PAL genes were identified and by compared the typical genes that participate in phenylalanine metabolic pathways. For example, LOC_Os06g44620.1 (Os4CL4), which encodes a 4-coumarate:coenzyme A ligase (4CL) and is thought to be involved in phenylalanine biosynthesis (Yoon et al., 2015), was more highly expressed in TeQing leaves than in Lemont leaves. The results suggest that PAL metabolism may play important roles at the initial infection stage. In addition, 3-dehydroquinate synthase, shikimate kinase, prephenate dehydratase and anthranilate synthase component I-1 were also found from this metabolic pathway. As shown in the current study, the phenylalanine pathway was up-regulated in TeQing leaves in the early stage (12 h) after AG1 IA infection. We speculated that at the initial stage of infection, TeQing leaves may prevent the encroachment of pathogens and protect the host cells by enhancing cell wall lignification and increasing other secondary metabolites. The difference between susceptible and resistant plants is associated with the ability of the plant to initiate defense processes effectively and in a timely manner. This is likely one of the reasons why the TeQing cultivar exhibits resistance to AG1 IA infection. Further research into these genes may provide candidates for the genetic improvement of rice and new insights into disease resistance.

### Changes in the JA and SA Pathways Related to AG1 IA Inoculation

Jasmonic acid, a linolenic acid-derived cyclopentanone, plays regulatory roles in plant development and responses to fungal infection (Pieterse et al., 2009). Research has shown that JA signaling activates resistance to necrotrophic pathogens (Yang et al., 2013). Treating rice plants with JA induced the expression of a series of PR genes, suggesting that JA is involved in rice immunity to pathogens (Simmons et al., 1992; Mei et al., 2006). Our results indicated that the JA pathway is involved in the disease resistance to AG1 IA, which is in line with previous reports showing that the JA pathway mediates plant resistance to pathogens (Matić et al., 2016). In addition, as the infection time increased, genes involved in alpha-linolenic acid metabolism significantly enriched in Lemont leaves after 24 h, and their expression suggested that the JA signaling pathway activation in response to AG1 IA is stronger in Lemont leaves. Furthermore, because JA synthesis-related genes were up-regulated at 12 h, we speculate that the involvement of JA in the rice immune response may occur at the early stage of infection. The plant hormone SA is an important endogenous signal molecule in plant defense responses (Vlot et al., 2009). The SA response pathway is thought to be effective against biotrophic pathogens (not strictly distinguished) (Glazebrook, 2005). As important signal molecules in the plant defense response, SA- and JA-mediated pathways are relatively independent. However, SA–JA cross talk can also be a powerful mechanism used to prioritize one pathway over the other, depending on the sequence of events and type of threat encountered (Pieterse et al., 2012). We speculate that parasitic processes occur in the early stage of infection and, from the expression level of OsICS1, that SA is also involved in disease resistance.

## Conclusion

In summary, the gene expression patterns in TeQing and Lemont leaves after AG1 IA infection provide a comprehensive overview of the transcriptome of two rice cultivars with contrasting disease responses. These patterns highlight the transcriptional variations between control and AG1 IA-infected plants. AG1 IA activated multiple resistance pathways, and DEGs were involved in the defense response, signal transduction and other processes, suggesting that pathogen response is regulated by multi-gene networks. A series of genes involved in disease-related metabolic pathways were significantly regulated, demonstrating that AG1 IA infection negatively affects the growth of rice. In addition, we identified some metabolic pathways that were common to both the TeQing and Lemont cultivars. We believe that the biosynthesis of JA, the basic plant metabolic processes of photosynthesis and photorespiration, and the synthesis of phenylalanine compounds may be important for disease tolerance in rice. Further research is necessary to identify candidate genes to determine whether these DEGs are necessary for the differences associated with AG1 IA infection between TeQing and Lemont plants. The data obtained in this study may be used to identify the most suitable candidate genes for carrying out genetic modification of susceptible rice cultivars to generate disease-tolerant rice cultivars.

## Availability of Supporting Data

The raw sequence data supporting the results of this article are available in the Short Read Archive (SRA) (accession number SRP113646). https://www.ncbi.nlm.nih.gov/Traces/study/?acc=SRP113646

## Author Contributions

JiZ completed the majority of the work for this article, including the experimental design, data analysis, and drafting of the manuscript. LC and CF provided useful suggestions. JiZ and YC carried out bioinformatic analyses. PL, LW, HL, SL, QD, SW, JuZ, YL, and AZ revised the manuscript. All authors read and approved the final manuscript.

## Conflict of Interest Statement

The authors declare that the research was conducted in the absence of any commercial or financial relationships that could be construed as a potential conflict of interest.

## Footnotes

1 http://cole-trapnell-lab.github.io/cufflinks/

2 http://ccb.jhu.edu/software/tophat/index.shtml

3 http://bowtie-bio.sourceforge.net/

4 http://rice.plantbiology.msu.edu/

5 http://rice.plantbiology.msu.edu/

6 http://geneontology.org/

7 http://www.genome.jp/kegg/

8 http://www.ricedata.cn
