# Supplementary material for: Comparative Transcriptome Analyses of Gene Expression Changes Triggered by Rhizoctonia solani AG1 IA Infection in Resistant and Susceptible Rice Varieties
Source: Front Plant Sci. 2017 Aug 17;8:1422. doi: 10.3389/fpls.2017.01422 (PMC5562724; doi:10.3389/fpls.2017.01422)
Supplement: Supplementary file 9 [file Data_Sheet_1.DOCX]

Figure S1-264214_Zhang_Image 1.JPEG. A Schematic representation of sheath blight inoculation; B Symptoms of sheath blight disease detected in TeQing and Lemont (The figure below).

Figure S2-264214_Zhang_Image 2.JPEG. Infection of hypha in inoculated leaves at different time point.

Figure S3-264214_Zhang_Image 3.JPEG. The overall relatedness of transcriptomes of different times.

Figure S4-264214_Zhang_Image 4.JPEG. Assess the similarity between samples across conditions by PCA analyse.

Figure S5-264214_Zhang_Image 5.JPEG. Functional classification of different express genes (DEGs) in the Teqing (A) and Lemont (B).

Figure S6-264214_Zhang_Image 6.PDF. Verification of differentially expressed genes by qRT-PCR.

Supplementary Table 1. Specific primers of differential gene sequences for qRT-PCR.

Supplementary Table 2A. Statistics of Illumina sequencing data; 2B. Summary for clean reads mapping to the Oryza sativa Nipponbare reference genome.

Supplementary Table 3. The 4802 co- regulated genes in both rice cultivars at different Time Points(L for Lemont;T for TeQing).

Supplementary Table 4. Functional classification of different express genes (DEG) in each clusters for the “Teqing” .

Supplementary Table 5. Functional classification of different express genes (DEG) in each clusters for the “Lemont”.

Supplementary Table 6. Analysis of pathways involving up-regulated genes after AG1 IA inoculation in TeQing.

Supplementary Table 7. Analysis of pathways involving up-regulated genes genes after AG1 IA inoculation in Lemont.

Supplementary Table 8. DEGs assosite with Plant-pathogen interaction at 12 and 24 h.
