# Supplementary figures and images for: Comparative Transcriptome Analyses of Gene Expression Changes Triggered by Rhizoctonia solani AG1 IA Infection in Resistant and Susceptible Rice Varieties
Source: Front Plant Sci. 2017 Aug 17;8:1422. doi: 10.3389/fpls.2017.01422 (PMC5562724; doi:10.3389/fpls.2017.01422)

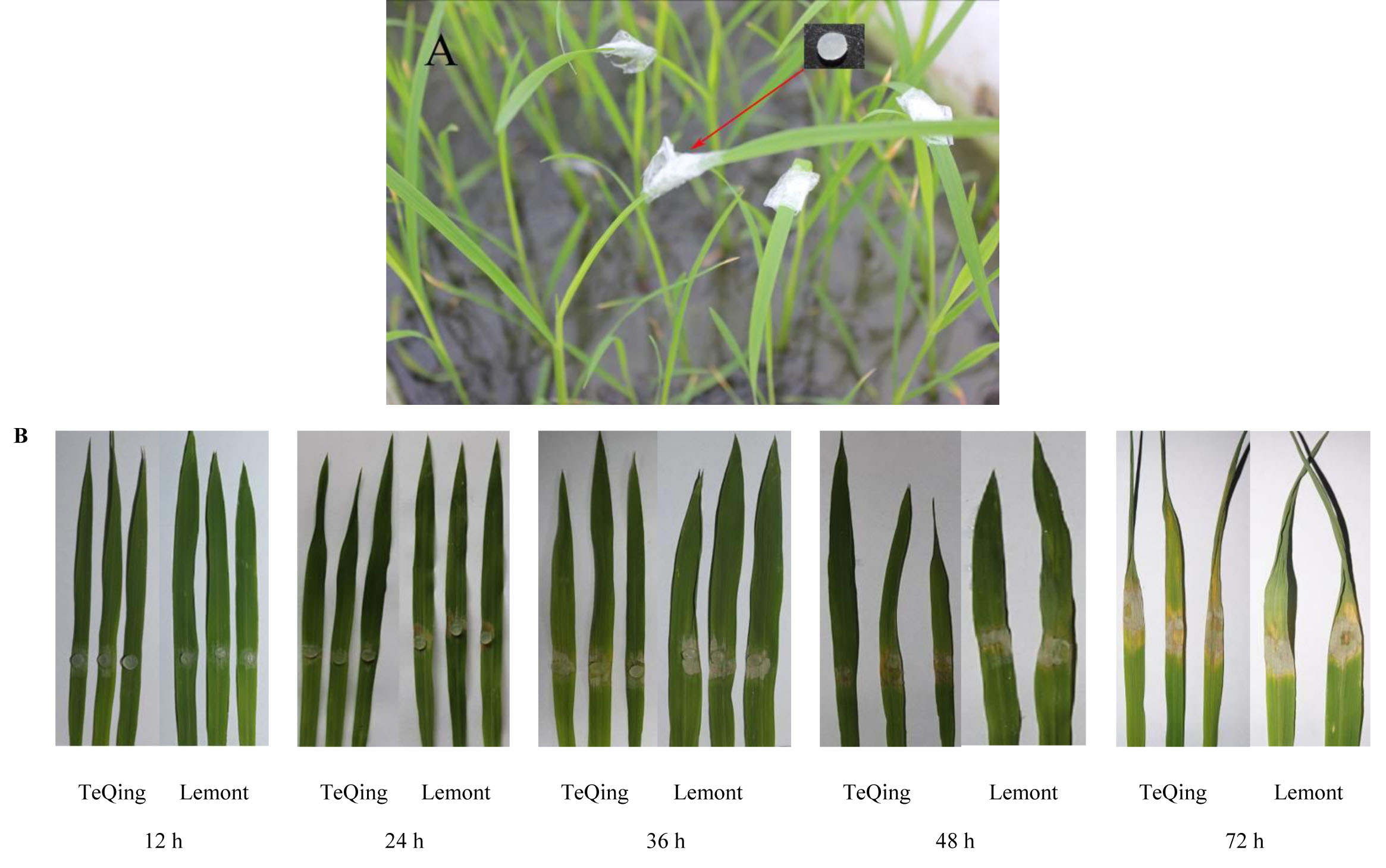

Supplement: Supplementary file 10 [file Image_1.JPEG]

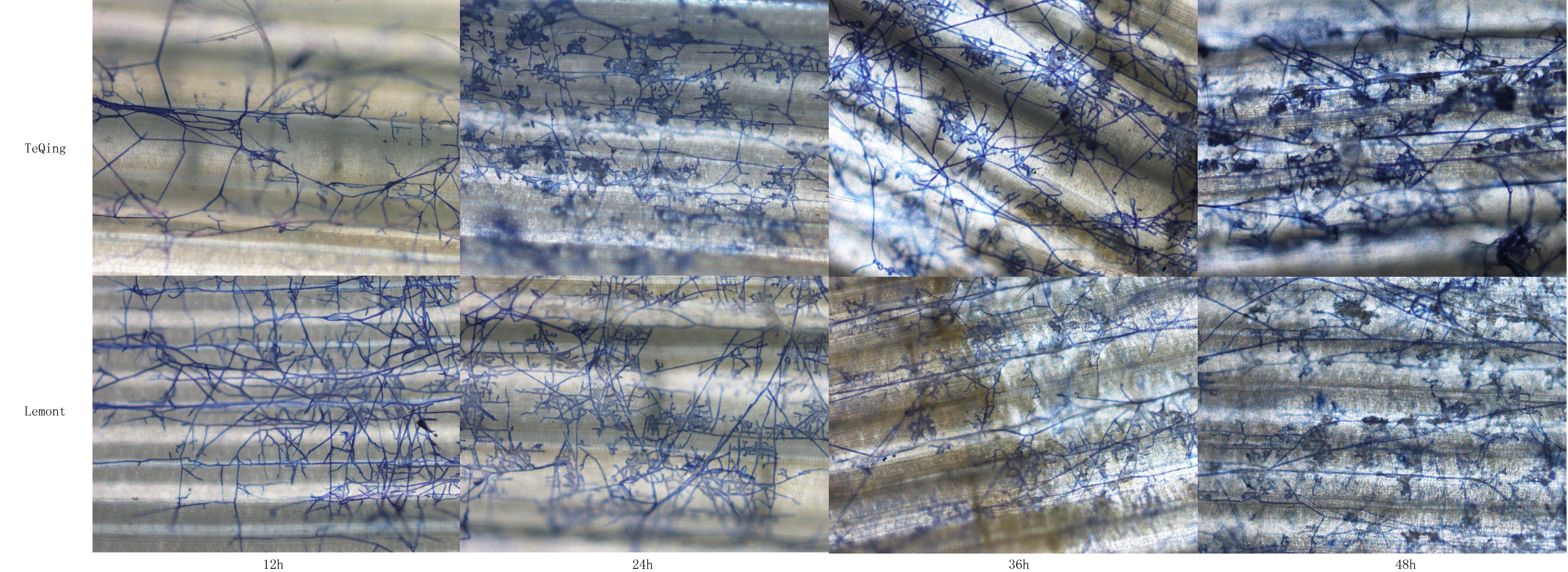

Supplement: Supplementary file 11 [file Image_2.JPEG]

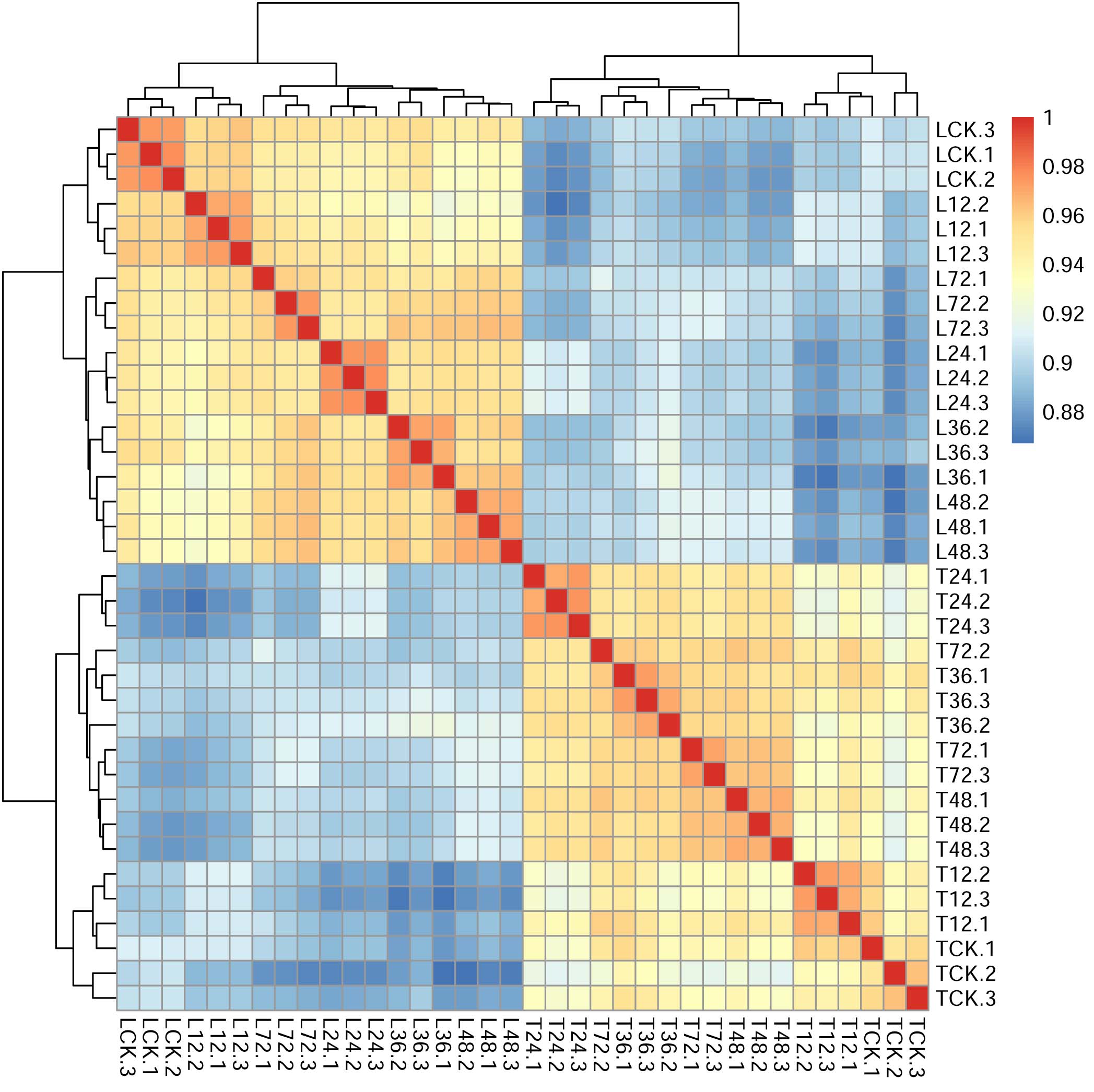

Supplement: Supplementary file 12 [file Image_3.JPEG]

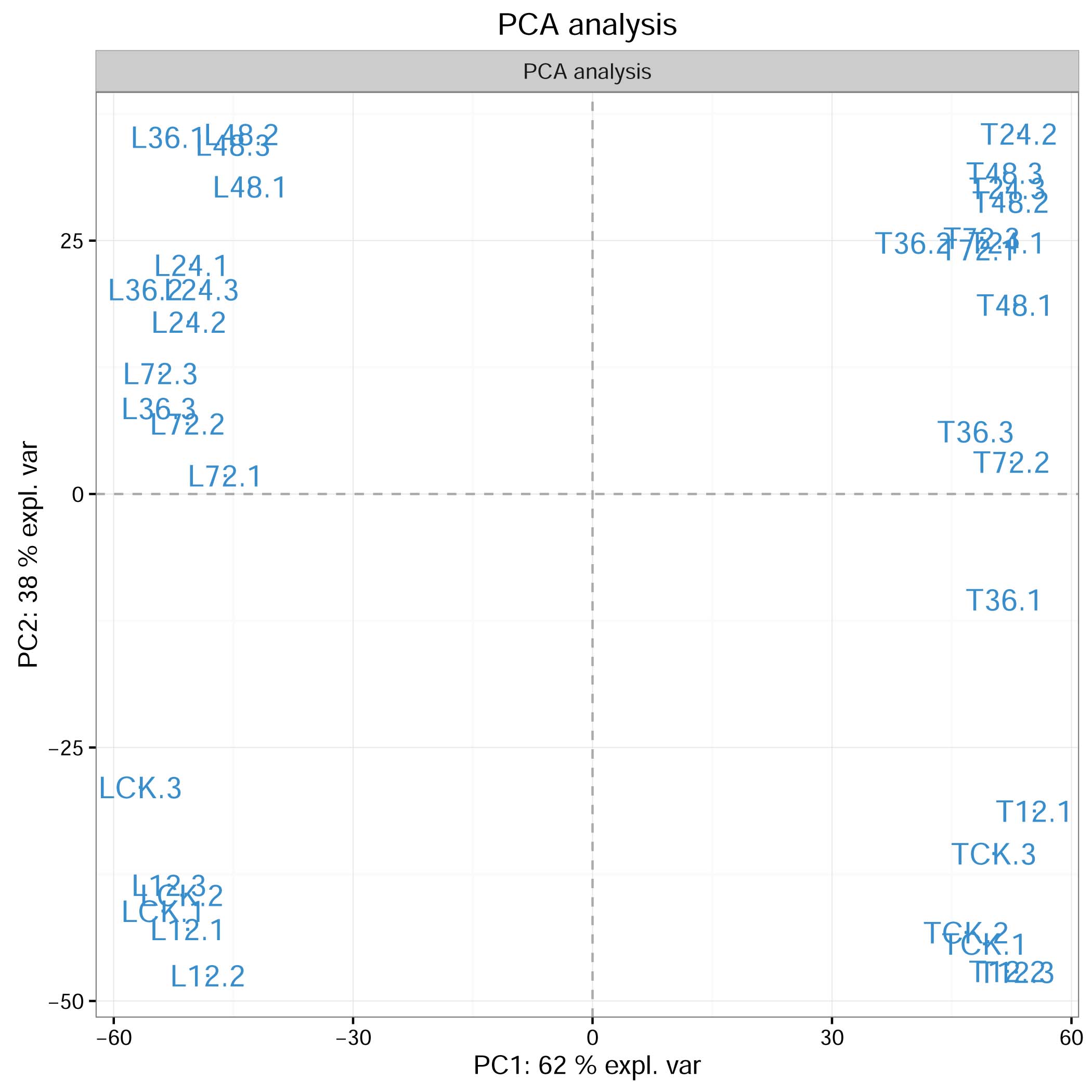

Supplement: Supplementary file 13 [file Image_4.JPEG]

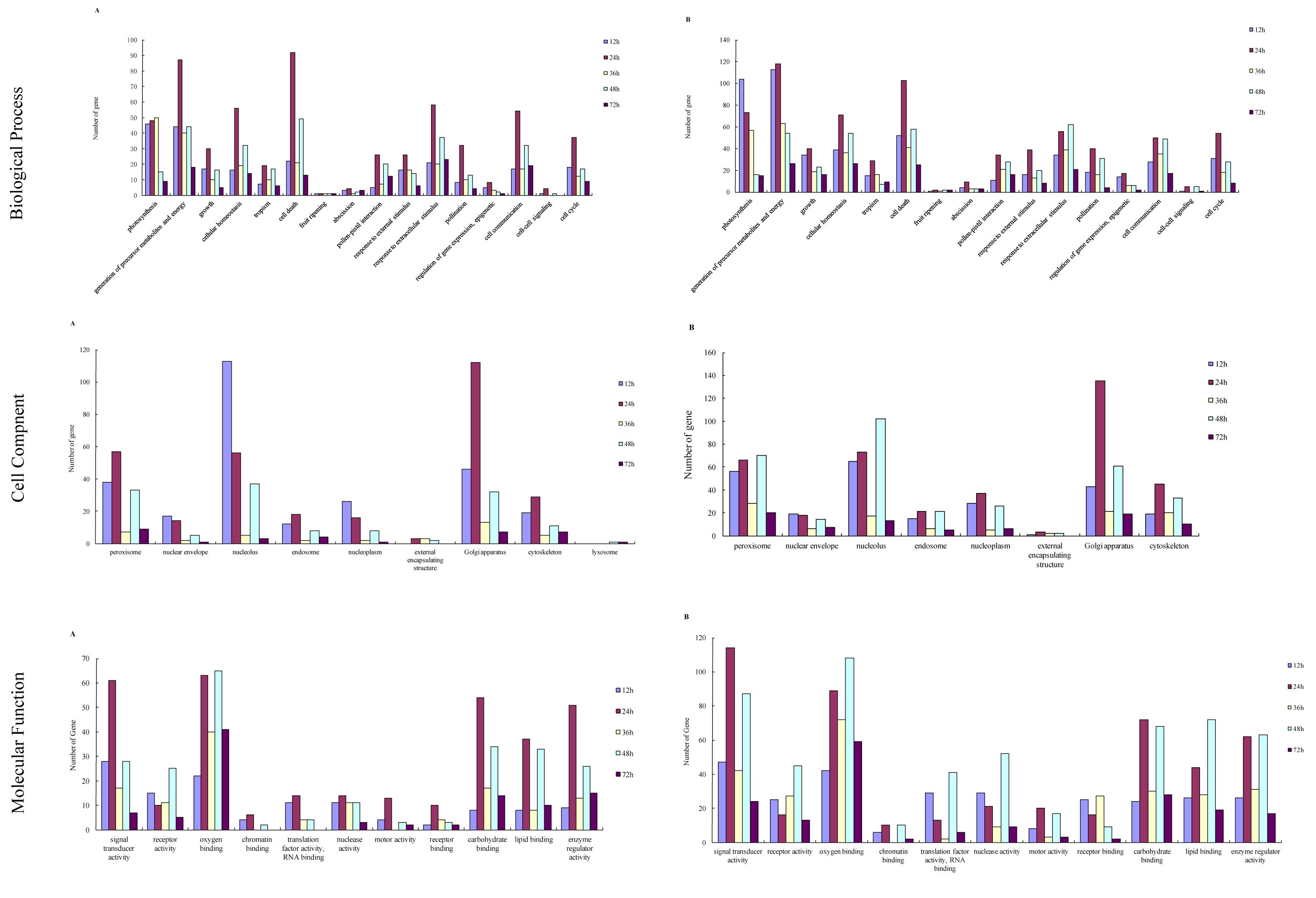

Supplement: Supplementary file 14 [file Image_5.JPEG]
