# Supplementary material for: Comparative Transcriptome Analyses of Gene Expression Changes Triggered by Rhizoctonia solani AG1 IA Infection in Resistant and Susceptible Rice Varieties
Source: Front Plant Sci. 2017 Aug 17;8:1422. doi: 10.3389/fpls.2017.01422 (PMC5562724; doi:10.3389/fpls.2017.01422)
Supplement: Supplementary file 15 [file Image_6.PDF]

Figure S6. RT-qPCR validation of parts of differentially expressed genes identified by Illumina sequencing. Histogram: Relative expression, detection results of real-time fluorescent quantitative PCR; Line graph: log2FC, fold change in differential expression genes in the transcriptome.

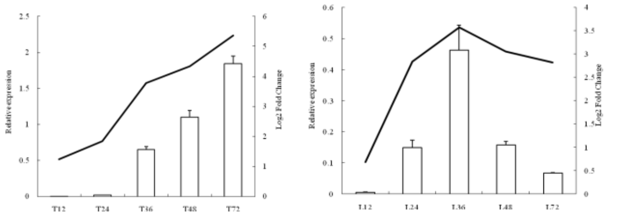


LOC_Os11g47600.1

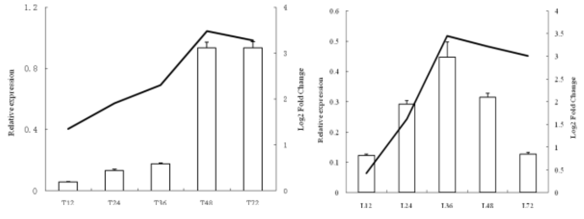


LOC_Os12g43380.1

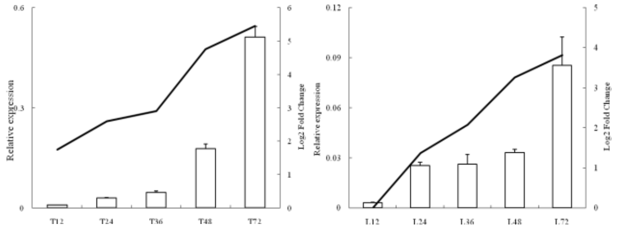


LOC_Os10g28080.1

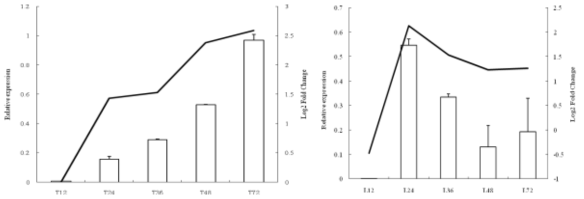


LOC_Os11g29870.1

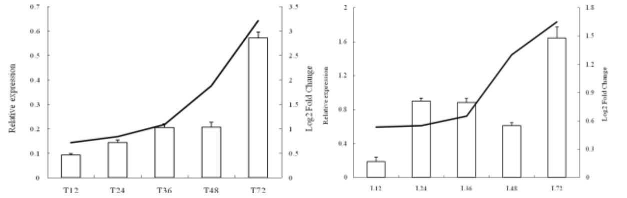


LOC_Os01g47070.1

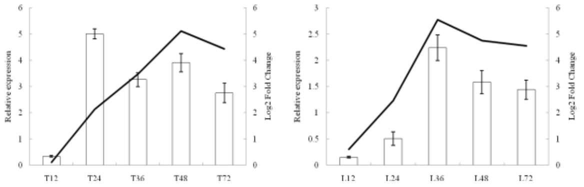


LOC_Os12g36880.1

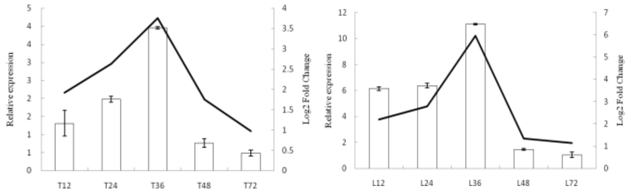


LOC_Os08g39840.1

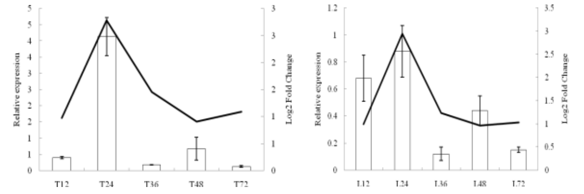


LOC_Os02g08500.1

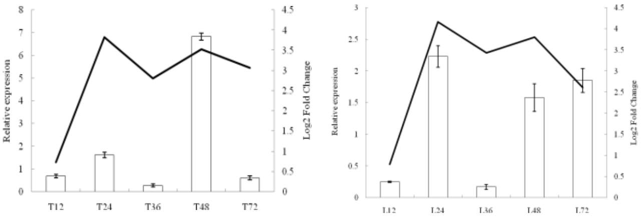


LOC_Os01g42410.1

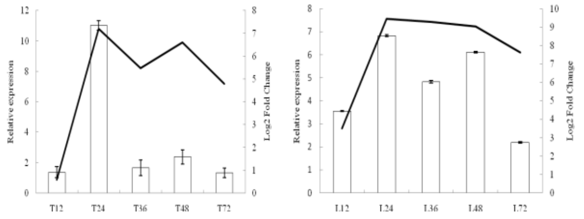


LOC_Os11g32650.1

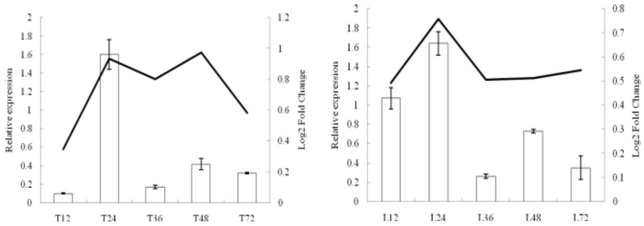


LOC_Os01g42380.1

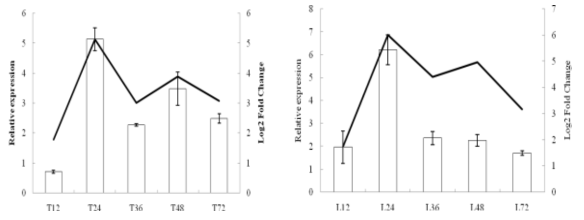


LOC_Os02g41680.1

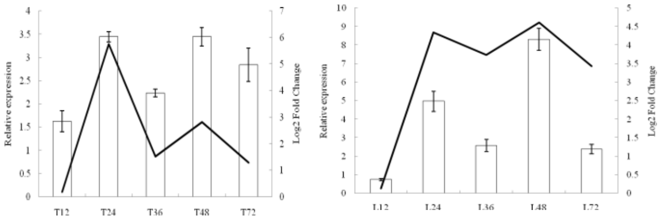


LOC_Os03g12500.1

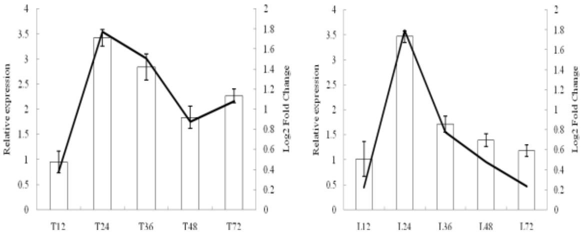


LOC_Os01g09800.1
